# Implementation of an Electronic Ionosonde to Monitor the Earth’s Ionosphere via a Projected Column through USRP

**DOI:** 10.3390/s17050946

**Published:** 2017-04-25

**Authors:** Jhon Jairo Barona Mendoza, Carlos Fernando Quiroga Ruiz, Carlos Rafael Pinedo Jaramillo

**Affiliations:** 1 Geopositioning Laboratory, Universidad Del Valle, Cali 760036, Colombia; carlos.quiroga@correounivalle.edu.co; 2 ETSI Universidad Politécnica de Madrid, Madrid 28040, Spain; 3 School of Electrical and Electronic Engineering, Universidad Del Valle, Cali 760036, Colombia; carlos.pinedo@correounivalle.edu.co

**Keywords:** ionosonde, ionosphere, radar, SDR, USRP

## Abstract

This document illustrates the processes carried out for the construction of an ionospheric sensor or ionosonde, from a universal software radio peripheral (USRP), and its programming using GNU-Radio and MATLAB. The development involved the in-depth study of the characteristics of the ionosphere, to apply the corresponding mathematical models used in the radar-like pulse compression technique and matched filters, among others. The sensor operates by firing electromagnetic waves in a frequency sweep, which are reflected against the ionosphere and are received on its return by the receiver of the instrument, which calculates the reflection height through the signal offset. From this information and a series of calculations, the electron density of the terrestrial ionosphere could be obtained. Improving the SNR of received echoes reduces the transmission power to a maximum of 400 W. The resolution associated with the bandwidth of the signal used is approximately 5 km, but this can be improved, taking advantage of the fact that the daughterboards used in the USRP allow a higher sampling frequency than the one used in the design of this experiment.

## 1. Introduction

The development of communication technologies and new proposals for the use of electromagnetic wave propagating in space have brought important studies on the role played by the ionosphere in these processes [1]. The ionosphere is the region of the terrestrial atmosphere located between approximately 60 and 500 km in altitude; however, these values are not absolute since they vary according to factors, like time of day, time of year, solar cycle and any factor that varies the emission of X-rays and ultraviolet (UV) radiation coming from the Sun. These are also the main causes for the existence of this region in the high atmosphere, as well as the terrestrial electric and magnetic fields. Some of the main attributes of this region are that it is composed of plasma, contains large numbers of protons and free electrons, besides neutral gases, which results in variable electromagnetic conditions throughout the day that act as a disturbing medium to the electromagnetic waves that manage to cross it (this being an unwanted effect), while acting as a reflection screen for those that cannot cross it. These characteristics disturb the expected results of the Global Navigation Satellite System (GNSS) and of several telecommunications applications.

Research and study of the ionosphere has led to the development of new technologies, such as ionosondes, associated with complex monitoring systems in order to observe, estimate and model this atmospheric layer. The study of the ionosphere implies a technological advance of great impact for the reasons previously mentioned [2].

The evolution of instrumentation for the study of the ionosphere has been linked to advances in digital techniques since the 1970s. Analog ionosondes began to be replaced by digital instruments and custom computer circuits to perform digital integration and digital spectrum analysis. This article details an approach to the conception, design and implementation of a vertical ionosonde by means of software-defined radio (SDR) technology using a universal software radio peripheral (USRP) unit, which allows the estimation of the different heights of the layers of the ionosphere and facilitates its study.

This type of low-cost instrument is an implementation of modern technologies, preceded by other technological developments. Within the instrumental development for the low-cost ionospheric sounding, there are works such as the Digisonde Portable Sounder (DPS) by Reinisch and Haines [3], the Low-Cost Ionosonde System by Stamper and Davis [4] and the Digisonde-4D being the latest digital ionosonde that the University of Massachusetts Lowell Center for Atmospheric Research (UMLCAR) developed during 2004–2008; while preserving the basic principles of the Digisonde family, the Digisonde-4D model introduces a number of important hardware and software changes that implement the latest capabilities of new digital RF circuitry and embedded computers [5], among many others. All of these, although some of a significant efficiency, correspond to instruments with technologies prior to the development of the universal software radio peripheral (USRP), differentiated by the lack of flexibility in data processing, greater rigidity of hardware structures and a generally smaller data transmission channel. Among other comparable developments is Vierinen [6] and Dautbegovic [7], based also on SDR technologies with USRP differentiated by some different hardware elements and the use of a pulse modulation technique Frecuency Modulated Continuous wave (FMCW) or chirp.

This work was motivated from the knowledge acquired by the authors in the First Ionosonde School In The Radio Observatory of Jicamarca (Lima District, Peru) in the year 2013.

## 2. Ionospheric Characterization and Data for Specification

This section describes the main characteristics of the ionosphere and how it influences electromagnetic waves.

### 2.1. Ionosphere and Phenomena

The terrestrial atmosphere is constituted by several regions, one of them being the ionosphere, which is located in the upper atmosphere between approximately 80 km and outer space [8] (although authors differ in the dimensions of this layer). This region is composed of partially-ionized gas that behaves as an interface between the terrestrial habitable zone and outer space. The ionosphere is exposed to solar radiation and other phenomena, such as terrestrial magnetism, solar winds and magnetic storms, which generate ionization and photo-dissociation processes [9]. These two processes occur simultaneously and are implicitly caused by the absorption of the energy of X-rays and ultraviolet rays from the Sun by molecules like NO, H_2_, O, H, He; the first process leads to a large number of electrons breaking the bonds they maintain with the molecular nucleus, resulting in free electrons and positive ions, while the second process leads to molecule dissociation. These processes depend directly on the molecules upon which radiation falls, the energy carried by radiation (which varies with the solar cycle) and on geographic location, among others, causing the terrestrial ionosphere to be highly variable. Recombination and molecular association are two inverse processes to ionization and photo-dissociation, which depend in equal measure on the atmospheric density that varies with height. The recombination phenomenon reintegrates the free electrons with the positive ions, establishing the original equilibrium of the molecules, and the molecular association establishes the existing relationships between photo-dissociated molecules and the surrounding molecules, thus creating new molecules.

**Figure 1:**
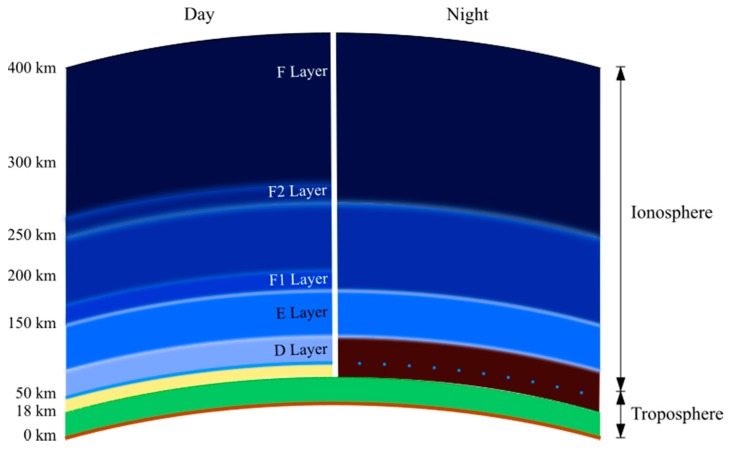
Ionospheric layers.

Ionization and photo-dissociation are accentuated by day when the Sun’s rays arrive with more intensity over the ionosphere. When night falls, recombination and molecular association predominate due to the low amount of incoming energy. These energy balance phenomena create the ionospheric layers D, E, F1 and F2, which are variable in time, and its greater disturbing factor is the solar activity presented [8], as shown in Figure 1. The ionosphere, due to the ionization phenomenon present in it, is capable of reflecting the incident high frequency (HF) electromagnetic signals (3–30 MHz), allowing the determination of its height by means of radar.

### 2.2. Basic Propagation Principles in the Ionosphere

Free electrons in the ionosphere affect the electromagnetic waves traveling through it at a frequency of 3 kHz–30 GHz. These waves alter the movement of free electrons and, depending on the amount of electrons, their range of motion, frequency and amplitude of the incident wave; they can influence the waves causing total absorption, reflection or phase delays. The amount of energy that is refracted, reflected, transmitted or absorbed from the electromagnetic wave can be determined by comparing the incident wave and resonance frequency or plasma frequency.

Plasmas are not rigid structures. The electromagnetic force creates the organization of a collection of charged particles. Once an electron is displaced from the uniform organization of ions, due to the alternate field of the incident electromagnetic wave, the displacement of the electron generates an electric field that attempts to restore the plasma’s neutrality by returning it to the original position. However, because the electron’s inertia is surpassed, it begins to oscillate in its position with a certain frequency known as the plasma frequency, *w_p_*. The relationship between electron density and plasma frequency is described in Equation (1):

(1) $$w_{p}=2\pi f_{p}=\;\sqrt{\frac{Ne^{2}}{\varepsilon_{0}m_{e}}}$$

*e* = 1.6021766208(98) × 10^−19^ C
*m_e_*= 9.10938356(11) × 10^−31^ kg
ε_0_ = 8.854187817 × 10^−12^ F/m

where *w_p_* is the angular plasma frequency, *f_p_* is the plasma frequency, *N* is the electron density, *e* is the electron’s charge, ε_0_ is the permittivity in the free space and *m_e_* is the electron’s mass.

Plasma frequency plays a fundamental role in the refractive index and, consequently, in the propagation at the ionosphere. The refractive phase index in the ionosphere, *μ_p_*, is given in the simplest case by Equation (2) and illustrates some unique characteristics of propagation in ionized media compared to non-ionized media, such as those occurring in the lower atmosphere. It is assumed that there are not effects from the Earth’s magnetic field, neither in the presence of a neutral atmosphere, nor from the ions in which the free electrons are absorbed. In reality, these effects cause radio wave distortion when they encounter the ionosphere:

(2) $$\mu_{p}=\frac{c}{v_{p}}=\sqrt{1-\frac{w_{p}^{2}}{w^{2}}}$$

where *w* is the angular frequency of the transmitted radio wave, *c* is the speed of light in a vacuum and *V_p_* is the radio wave’s phase velocity [10,11].

## 3. The Ionospheric Radar

**Figure 2:**
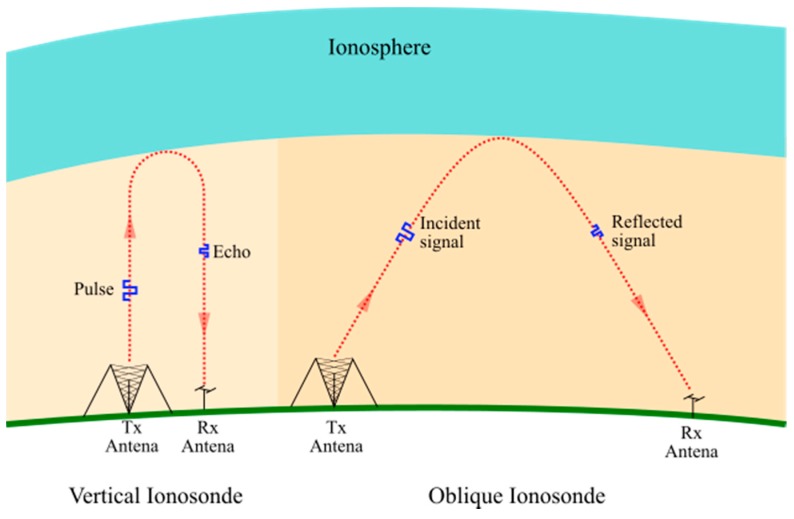
Vertical and oblique ionosondes.

At present, measurements associated with the height of the ionosphere are taken by two types of sounding, vertical and oblique. These operate under the same principles; however, the geometry of vertical sounding has a more direct interpretation than oblique sounding measurements presented in Figure 2.

An ionospheric radar or ionosonde is basically an HF radar that transmits electromagnetic pulses at different frequencies from a certain point on Earth towards the ionosphere and receives its echo at the same or another point on Earth. The signal sent to the ionosphere propagates to a height where the frequency of the radio wave *w* is equal to the frequency of the ionospheric plasma *w_p_*, causing a maximum reflection intensity at this point. By processing the echo measured between the emission and reception signals, it is possible to infer the height at which the reflection happens, although this is not the actual height, but one that is known as the virtual height (*h_v_*) that would occur if the signal sent had a speed equal to that of light in a vacuum, which constitutes a fundamental parameter in the construction of an ionospheric profile or ionogram [12].

### 3.1. Range, Resolution and Radar Equation

The ionosonde sends a train of pulses to the target, and with the time-delay of the echo Δ*t*, the range *R* is calculated from the transmission station to the distance at which each of the ionospheric layers are with respect to the Earth’s surface. This distance calculation is given by Equation (3):

(3) $$R=\frac{c\cdot \Delta t}{2};\;h_{v}=R$$

where *c* is the speed of light and *h_v_* is the virtual distance calculated for the ionospheric layers.

**Figure 3:**
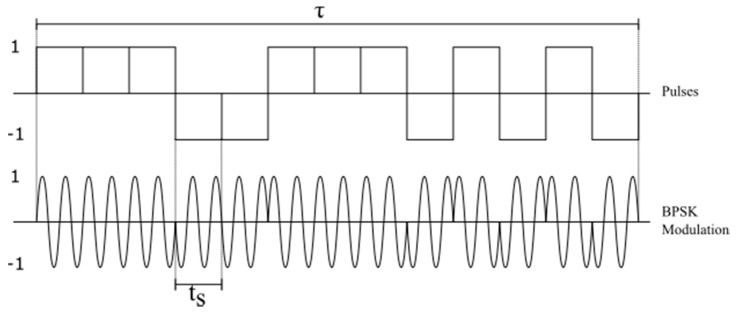
BPSK modulation.

The resolution ΔR (Equation (4)) is the minimum distance at which different ionospheric layers can be detected, as a function of the radar bandwidth *B* and the range *R* [13]:

(4) $$\Delta R=\frac{c\cdot t_{s}}{2}=\frac{c}{2\cdot B};\;B=\frac{1}{t_{s}}$$

where *t_s_* corresponds to the duration of the resulting radar pulse in the receiver, when applying the pulse compression technique to be explained in Section 3.2 and shown in Figure 3, and *B* corresponds to the inverse of *t_s_*.

The minimum detection range for investigation in the ionosphere is determined by the pulse width τ shown in Equation (5):

(5) $$R_{min}=\frac{c\cdot \tau }{2}$$

The maximum distance depends directly on the detection capacity of the system, as well as the radar’s pulse repetition frequency (PRF) or the pulse repetition time (PRT).

Studies of the ionosphere, fortunately, do not require strict specifications on integration and speed, so it becomes easy to work with the condition of the unambiguous range *R_u_* (Equation (6)) [14], which is the maximum distance at which a target is expected to be found.

(6) $$R_{u}=\frac{c}{2\cdot PRF};\;PRF=\frac{1}{PRT}=\frac{1}{\Delta t}$$

A guide for the design of a radar system is the radar equation, which estimates the range according to the desired characteristics presented in Equation (7) [15]:

(7) $$P_{r}=\frac{P_{t}\cdot G_{t}}{4\pi \cdot R^{2}}\cdot \frac{\sigma }{4\pi \cdot R^{2}}\cdot A_{e}$$

where *P_r_* is the power received, the first factor of Equation (7) is the power density received at a distance R from the radar that radiates a power *P_t_* from the antenna with a gain *G_t_*; in the second factor, σ is the radar cross-section (RCS), in the denominator, is the divergence of the echo of the signal on its return to the radar; and finally, *A_e_* is the effective area of the receiving antenna, which collects a part of the echo power that returns to the radar.

Taking into account the considerations of the radar equation, and if the maximum radar range is defined, *R_max_*, when the received signal is equal to the minimum detectable signal, *S_min_* [15], Equation (8) is obtained:

(8) $$R_{max}^{4}=\frac{P_{t}\cdot G_{t}\cdot A_{e}\cdot \sigma }{{\left(4\pi \right)}^{2}\cdot S_{min}}$$

From these statements, it can be said that pulses of short duration are required to obtain high resolution, and to achieve the detection of distant targets, a high power peak pulse, *P_t_*, a long pulse duration, τ, or a small PRF is required.

### 3.2. Pulse Compression and BPSK

The pulse compression technique is developed because of the energy cost caused by short-duration pulses and the great power required for a good detection range and resolution [12]. This technique allows sending a high average power in a long pulse, with the resolution of a narrow pulse [16], which makes it advantageous. However, this technique is more complex on the generation of pulses in the transmitter and target detection, which requires mathematical pre- and post-processing [17]. It essentially consists of widening or stretching a pulse by a given factor, using correctly-encoded radar pulses [18], thus improving the signal to noise ratio (SNR) of the output and input.

Spread spectrum-based techniques, such as binary phase-shift keying (BPSK) or linear frequency modulation (LFM), are used for pulse compression. BPSK modulation is ideal for the design of an ionosonde and to encode transmitted signals because its autocorrelation function has a low level of sidelobes in time, decreases the peak power of the transmitted wave, increases the average power, as well as provides easy implementation in both the transmitter and the receiver. With this, the pulse duration τ is subdivided into an integer of sub-pulses (chip or code bit) of duration *t_s_*, with an amplitude being zero or one where the phase is constant and varies between zero and π radians depending on the code used, as shown in Figure 3.

After the modulation process, the signal is amplified and transmitted through the medium, and a small fraction is reflected, where the echo or the replica has the same characteristics of the transmitted wave, apart from the amplitude and added noise. Once the decoding of the received wave is done, it is possible to obtain all of the information contained by signal processing [19].

There are different bit code sequences that fulfil a good autocorrelation function and low levels of their sidelobes, such as Barker codes and complementary codes. To objectively validate whether the self-correlation function presents a good side lobe response compared to another, the expression of peak side lobes (PSL) (Equation (9)) is used [20].

(9) $$PSL=10log\left[\frac{\max\left(d_{m}{}^{2}\right)}{d_{0}{}^{2}}\right]$$

where $d_{m}$ is the level of lateral lobes and $d_{0}$ is the amplitude level of the main lobe.

According to Portieles and de Armas [21], the PSL calculations in 13-bit Barker codes and eight-bit complementary codes are −22.28 dB and −344 dB, shown in Equations (10) and (11):

“Theoretically in the complementary codes, the lateral lobe level is always zero for any length of code, although in practice that level of amplitude may be around 10^−16^ [21]”.

For Barker 13-bit code:

(10) $$PSL=10log\left[\frac{\max\left(1^{2}\right)}{13^{2}}\right]\approx -22,28\;dB$$

For eight-bit complementary code:

(11) $$PSL=10log\left[\frac{\max\left(10^{-16}{}^{2}\right)}{16^{2}}\right]\approx -344\;dB$$

demonstrating that the complementary codes present a better behavior than the Barker with respect to sidelobes. For this reason the complementary codes are used for the development of the ionosonde.

### 3.3. Matched Filter

A matched filter is the most efficient filter for discriminating between white Gaussian noise and received radar echoes. The main function of this type of filtering is to detect a signal where the frequency characteristic is known within a received signal.

**Figure 4:**
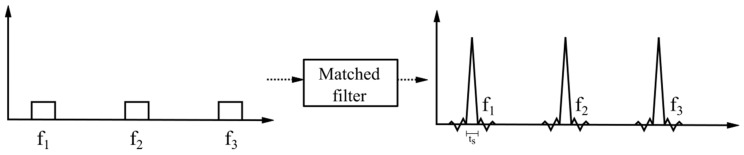
Matched filter for radar pulse compression.

This is based on the fact that two functions slide by, passing one over the other, where the sequence of correlations is calculated, which causes the elements of the distinctive signal to be recognized and compressed into a short pulse with a peak of width *t_s_* along with an intensity proportional to the echo received and some sidelobes. Figure 4 shows the relationship between transmitted pulses and compressed radar pulses [22]. The gain of the matched filter *M* implemented with finite impulse response (FIR) filters in terms of the SNR is expressed as shown in Equation (12):

(12) $$M=\frac{P_{2}/N_{2}}{P_{1}/N_{1}}$$

where *N*_1_ and *N*_2_ are the noise power at the input and output of the matched filter. Hence, the numerator and the denominator of the fraction are the output and input SNR, respectively.

It can be demonstrated that the gain of the matched filter, in terms of the SNR, is no more than the characteristic compression ratio in these filters given by Equation (13) and dependent on the pulse width and the bit code width.

(13) $$M=B\tau =\frac{\tau }{t_{s}};\;B=\frac{1}{t_{s}}$$

### 3.4. Integration of Pulses Received by the Radar

This consists of adding or integrating a quantity K of echoes received after N successive transmissions. The result of this operation raises the SNR in a single pulse, also integrating the noise, but due to its stochastic nature, the increase is generally smaller than the signal.

**Figure 5:**
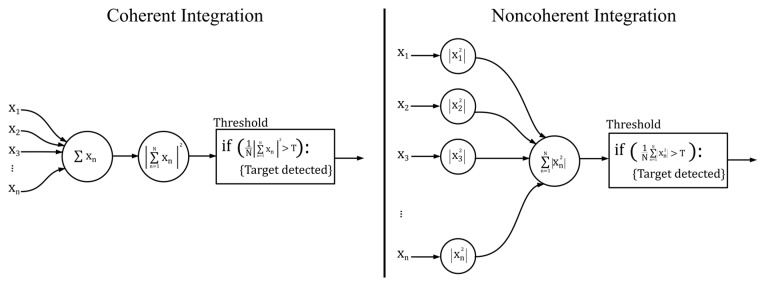
Comparison between the implementation of coherent and non-coherent integrations.

The pulse integration methods used are coherent and non-coherent, which have different results since they depend on the place where the signal processing chain is performed, as shown in Figure 5.

According to Zuccheretti and collaborators [14,23], the best integration method is the coherent one because the phase information is not lost and the SNR is reduced. Nevertheless, caution must be exercised with the number of integrations to prevent signal cancellation. On the other hand, non-coherent integration is not as efficient since it is not possible to use the mean zero noise value, which keeps the SNR constant, and phase information is lost. However, non-coherent integration is easy to implement and reduces the variance of the sum of the received noise, which increases the detection probability (*P_D_*) of targets. Regardless of the method chosen to perform the integration, *P_D_* is improved in relation to the noise in the signal.

### 3.5. System Losses and Noise Sources

Equation (7) has been described in its ideal form without taking into account the attenuation or power loss of the signal, which alters the transmitted pulses, and ignoring them produces errors in range estimation. Geometric attenuation is the most influential, with a variation of 80–120 dB, but comes with other secondary causes, such as the average ionospheric absorption (non-deviative), polarization by decoupling, deviative attenuation, system internal losses and layer shielding, among others. On the other hand, a signal degradation source in the HF band is the noise, which can be internal, environmental or man-made [14], with levels varying from −50 dBm–113 dBm. To limit these noise effects on the signal, it is recommended to connect the amplifier as close as possible to the receiving antenna, to filter the signal by limiting the incoming spectral components, and digitally implement the pulse compression technique explained above.

### 3.6. Detection Capacity

**Figure 6:**
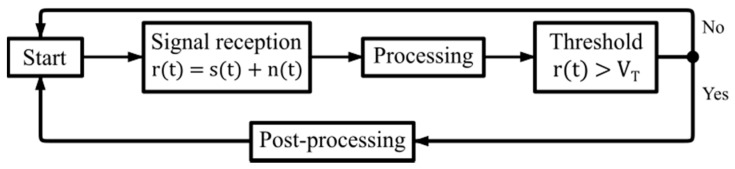
Algorithm for detection capacity at a radar receiver.

Determining whether or not an echo exists from the target can be made through decision-making, as shown by the algorithm in Figure 6.

**Figure 7:**
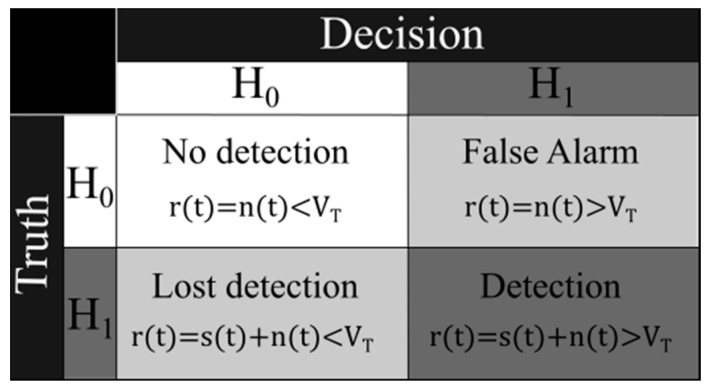
Combination of possible cases of detection.

The input signal at the receiver *r*(*t*) can be represented as the sum of the received pulse *s*(*t*) plus the additive white Gaussian noise (AWGN) *n*(*t*); after the mitigation processing of noise effects and attenuation, it must be determined if the signal *r*(*t*) contains an echo from an ionospheric layer. For that, the signal must exceed a threshold value *V_T_* that tests the existence *H*_1_ or nonexistence *H*_0_ hypothesis of the target. With *H*_1_ and *H*_0_, a probability table can be constructed taking into account that the process of detecting radar pulses is stochastic. For each measurement, there may be four cases, as shown in Figure 7.

Each of these hypotheses has a probability of occurrence; the probability of detection, which is defined as the probability of choosing *H*_1_ when *H*_1_ is true, and the probability of false alarm (*P_FA_*), which is defined as the probability of choosing *H*_1_ when *H*_0_ is true. According to the Neyman–Pearson criterion, it is sought to maximize *P_D_* for a specified *P_FA_* no greater than an alpha value of *P_FA_* ≤ α.

Mahafza in [13] presents the formal mathematical development for the *P_D_* and *P_FA_* probabilities arriving at the *P_D_*_(*PFA*, *SNR*)_ approximation shown in Equation (14):

(14) $$P_{D}\approx 0.5\;erfc\left(\sqrt{-ln\left(P_{FA}\right)}-\sqrt{SNR+5}\right)$$

where *erfc* is the function of the complementary error.

**Figure 8:**
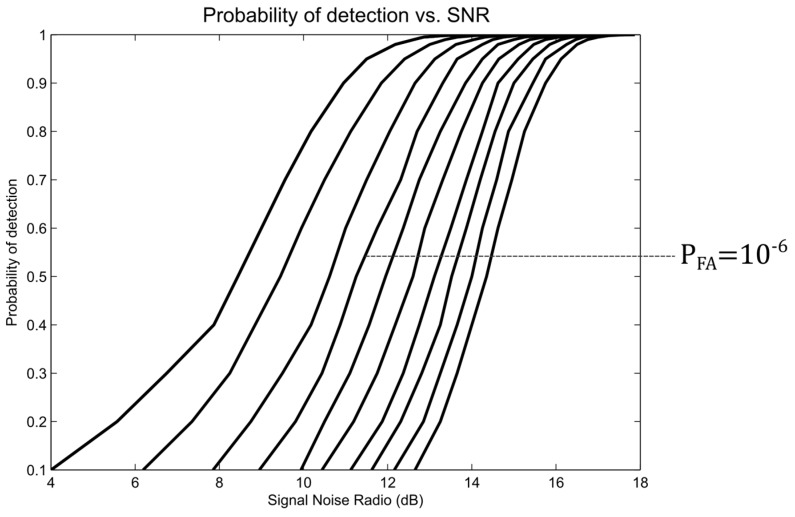
Probability of detection vs. SNR.

From Equation (14), the families of curves shown in Figure 8 are graphed. This graph is used for the design of the radars by setting the *P_FA_* and SNR values for a required *P_D_* [15]. Usually, only the possibility of altering the SNR to modify the detection capacity of the system is available, since *P_D_* and *P_FA_* are chosen according to the requirements. Designers usually choose a *P_FA_*= 10^−6^ [14].

## 4. Design of the System

**Figure 9:**
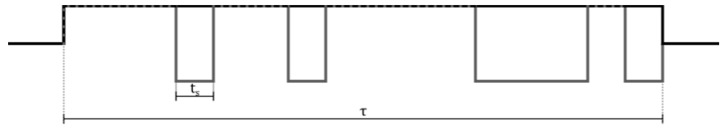
Waveform of a transmitted pulse. Complementary Code 1 of 16-bits.

**Figure 10:**
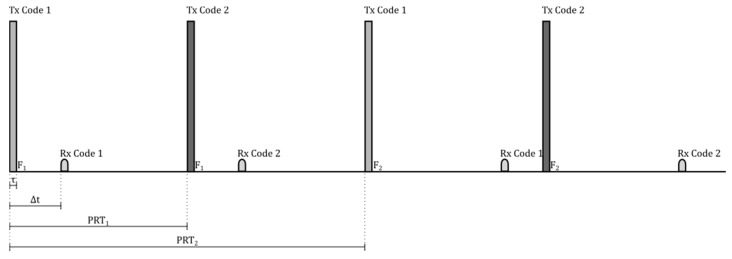
Transmitted and received pulses by the radar. PRT, pulse repetition time.

The design of the parameters of the transmitter and receiver of the ionosonde were made from the concepts presented in Section 2 and Section 3. Figure 9 shows the pulse shape with a duration of τ seconds that is transmitted by the radar, which is composed of a rectangular bit code sequence with a duration of *t_s_* seconds modulated in BPSK. Figure 10 shows the temporal behavior of the transmitted and received pulses that the ionosonde should have. The transmitter sends the pair of modulated complementary codes to an F_1_ frequency with a time between them of PRT_1_ and an echo reception time of Δ*t*. After PRT_2_ seconds have passed, the transmitter forwards the first code modulated to an F_2_ frequency, and thus, the process is repeated. It may be that after the PRT_2_ time passes, the code modulation frequency remains invariable due to the radar pulse integration parameter k.

In parallel, the receiver amplifies, filters, digitizes, demodulates and processes the captured echoes degraded by the influence of noise and losses to generate the maximum SNR. For this, the techniques of transferring to an intermediate frequency (IF), matched filters and digital down-conversion are mainly used, which allows the signal to be processed with two (I + jQ) channels, thus avoiding an average loss in the SNR of 3 dB (present if working with a single channel) [12].

Once the waveform of the transmitter, and what is expected at the receiver, is detailed together with the a priori knowledge detailed in the previous section, the parameters required at the radar are established. Table 1 shows the basic specifications that the prototype of the ionosonde must have.

From the equations detailed in Section 3.1 and the specifications in Section 2, a previous calculation of the parameters of the pulses to be transmitted shown in Table 2 is made. It must be taken into account that these are not the definitive values, but they serve to estimate other parameters.

On the other hand, Table 3 shows the maximum power of different ionosonde designs.

### 4.1. Receiver and Transmitter

**Figure 11:**
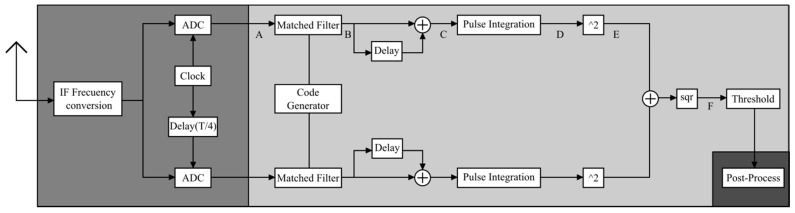
Block diagram of the digital receiver. IF, intermediate frequency.

Figure 11 shows that the hardware/USRP subsystem corresponds to the quadrature demodulation block where the RF/IF and IF/BB conversion is found, and the software subsystem corresponds to signal processing.

**Figure 12:**
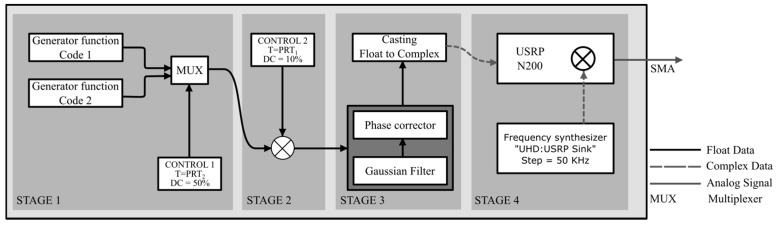
Transmitter programming scheme for pulse compression.

Figure 12 shows the design of the digital receiver. It is assumed that the input signal has already undergone a transfer process to the IF and a process of digital down-conversion (DDC) in the USRP from the daughterboards, leaving a complex demodulated digital signal.

In order to increase the maximum SNR, the code generator provides the binary sequence that is sent to the modulator of the transmitter and also to the matched filters so that a narrow pulse of low sidelobes can be obtained. Once the signal leaves the matched filter in the form of a pulse, it passes through a delay block so that the first code can be added to its complement. Then, the pulses are integrated using the coherent integration technique; the magnitude of this I/Q signal is calculated allowing the implementation of the threshold detection function. Finally, the post-processing of the signal is performed in which all of the mathematical operations necessary to calculate *h_v_* are performed.

With the block diagram of the digital receiver, all of the mathematical calculations of the different stages from the point F–B were carried out to guarantee the minimum SNR_F_, as well as the adjustment of the transmission signal parameters of Table 2. With the *P_FA_* and *P_D_* parameters of Table 1 and Figure 8, a minimum SNR_F_ of 11.25 dB was obtained. With this data, an SNR_B_ equal to 5.23 dB was found in the phase channel with minimum processing in terms of integrations (K = 2), assuming that the incoming signal has no complex components and all of the energy remains in the real part to determine that the minimum transmission power meets the established requirements [21].

(15) $$P_{t}L_{a}\tau =t_{s}P_{r}$$

Then, the minimum power required in the transmitter *P_t_* = 1.85 kW was obtained from the numerator of Equation (12), with a noise N2 = −60 dBm, and Equation (15) described in [17] with an attenuation *L_a_* = −130 dB [14] to maintain the specified SNR. It is then observed that, in order to obtain an SNR_F_, considering the highest possible noise, the transmission power is approximately 2 kW, which is an acceptable result when compared to the IPS-42 ionosonde. Nevertheless, considering that the minimum gain parameters were used in the receiver blocks, new parameters are set to lower the transmission power, this way redesigning the parameters of the pulses sent shown in Table 4.

In the Radar Handbook [15], the procedure to construct the complementary 16-bit codes used is detailed:

Cod 1= [1 1 1 −1 1 1 −1 1 1 1 1 −1 −1 −1 −1 −1], corresponding to Tx Code 1 in Figure 10.

Cod 2= [1 1 1 −1 1 1 −1 −1 −1 −1 −1 1 1 1 −1 1], corresponding to Tx Code 2 in Figure 10.

Table 3 allows establishing a transmitter with a peak pulse power of 400 W with a bandwidth of 1–20 MHz, which reduces the initial transmission power by 78%. From Table 4, and with the same attenuation and noise parameters, the gain factors in the receiver are obtained by an inverse process so that the initial SNR condition is satisfied, obtaining a total receiver gain of 25.25 dB. With this, an integration factor of K = 10 is chosen; the gain of the complementary codes is C = 2; and a gain of the matched filter of 128, which would exceed the estimated minimum value for the gain of the receiver.

Taking information from the previous tables, Table 5 is constructed where the estimation of the final parameters for the ionosonde is shown [2,14,21].

### 4.2. Power Amplifier and Antennas

Both the transmission system and the antennas are not a major part of this development so only the minimum specifications will be given. The amplifier must operate in a range of 1–20 MHz type A with a peak power in linear conditions of 400 W and be able to minimize harmonics and spurious signals to the maximum to increase efficiency. The antennas must be able to work with good gain in the specified frequency range due to the attenuations presented in the channel, and the main radiation lobe must be directed upwards.

According to the Istituto Nazionale di Geofisica e Vulcanologia (INGV) of Italy, a simple solution is a rhombic antenna or a delta antenna, which is its equivalent by lobe and band composition. On the other hand, an inverted log periodic antenna (LPA) for transmission offers greater frequency and spatial characteristics, as well as a uniform vertical incidence in the ionosphere, optimizing the performance of the component [27]. For reception, a broadband dipole arrangement is traditionally used, which has good performance in practice if spaced up to a quarter of a wavelength above the ground at the highest operating frequency [28].

## 5. Implementation of the System

From the design of the transmitter and the receiver, the ionospheric radar with SDR technology was implemented using a USRP N200, manufactured by Ettus Research, (National Instruments Corp., 4600 Patrick Henry Dr, Santa Clara, CA, USA).

Since it is one of the devices with the highest performance in the product family, it complies with the response specifications in the frequency and power of the design and has a good balance between the purchase price and functionality compared to other families on the market. The choice of basic Tx and basic Rx daughterboards was made based on the frequency operation range and the bandwidth criteria.

A Dell computer is used with an Intel Core2 Duo CPU 6400@2.13 GHz × 2, 64-bit operating system GNU-Linux Ubuntu 14-04 LTS, 983.3 MB RAM and a gigabit Ethernet port. Regarding the software, GNU-Radio was used, since it is an open source program, and there is a greater variety of information when it is linked to USRP and MATLAB.

### 5.1. Transmitter

In Figure 12, a detailed analysis of the programming schedule of the programmed transmitter is shown (not including the power stage), which is composed of four stages: code generation, control, filtering and phase correction and frequency synthesis, where Stages 1, 2 and 3 are implemented in the host, while the latter is implemented directly in the USRP.

**Figure 13:**
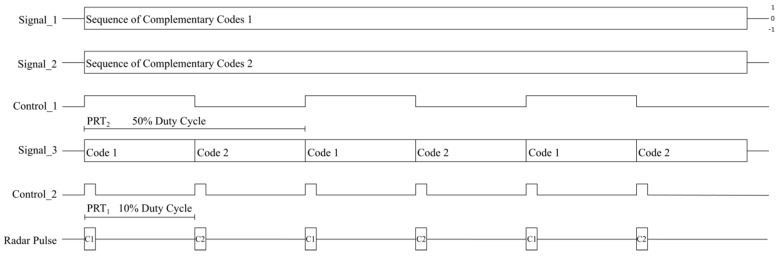
Conception of pulse generation with complementary 16-bit codes.

Figure 13 shows the way in which the code generating stage and control signals were implemented. Signal_1 and Signal_2 correspond to the code generating functions and are composed of a continuous succession of Complementary Codes 1 and 2, respectively. These signals enter a multiplexing block together with Signal Control_1, which has a period PRT_2_ with a duty cycle of 50%.

The output of the multiplexer is Signal_3, which is composed of a series of 160 bits corresponding to a sequence of 10-times code1 followed by another 160 bits of code2. Subsequently, Signal_3 passes through a code annulment block controlled by Control_2, which has a PRT_1_ period with a duty cycle of 10%, thus generating the 16-bit complementary codes.

With GNU-Radio, a block is programmed with Stages 1 and 2, which have Signal_1, Signal_2, Control_1 and Control_2 as input and ID, sampling rate, *t_s_*, τ, output amplitude and PRT_1_ as arguments, and handles float type data.

**Figure 14:**
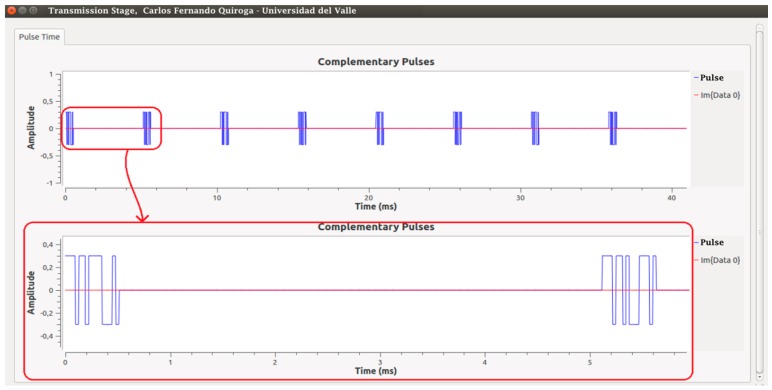
Output response from Stage 3 of the transmitter.

Stage 3, consisting of filtering and phase correction, is implemented before sending the signal in baseband (BB) to the USRP, where the high frequency components of the pulses are removed by means of a unity gain Gaussian filter. This filter generates a delay in the output with respect to the input wave, which is why a phase corrector is implemented and data casting from float to complex type is done. The result of this block is shown in Figure 14, where the baseband pulses are composed only of one real component. 

USRP Hardware Driver (UHD) is implemented for the last stage of frequency synthesis and the BPSK modulation. For this, the “UHD: USRP Sink” function is used, and the RF frequency enters the “Ch0: Center Freq” argument, with a range of 1–20 MHz and a 50-kHz-step. This change in frequency was implemented with a slider so that it changes manually in the GUI. This function of the UHD allows the cascaded integrator-comb (CIC) interpolator, the digital up-converter (DUC) and the output channels of the basic Tx daughterboard to be controlled in the FPGA.

**Figure 15:**
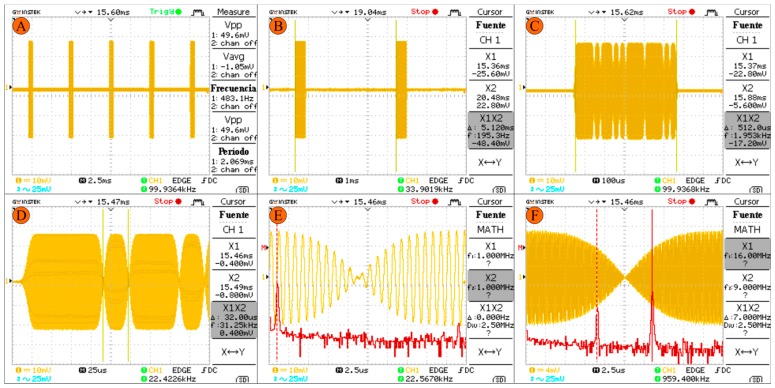
Transmitted pulses measured with the oscilloscope on the SubMiniature version A (SMA) connector of the RF front-end of transmission.

Figure 15 shows the results obtained in this stage to be coupled to the power. In (A) and (B), the pulses sent with PRT_1_ are presented. (C) shows the pulse with a duration of τ, and in (D) the duration of bit code *t_s_* is detailed. In (E) and (F), the fast Fourier transform is displayed for a frequency modulation of 1 MHz, 16 MHz and BPSK modulation. 

### 5.2. Receiver

**Figure 16:**
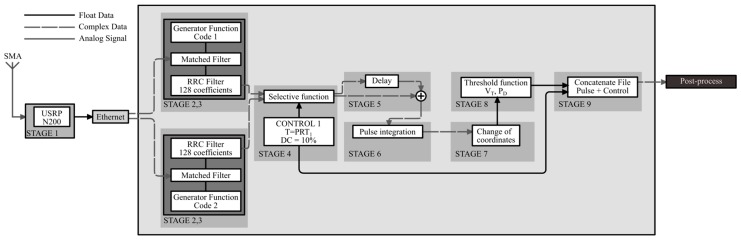
Receiver programming scheme. RRC, root raised cosine.

The scheme shown in Figure 11 was divided into nine stages, as shown in Figure 16.

With the already-demodulated waves, the digital signal processing was implemented in the GNU-Radio software subsystem to have a *P_D_* ≥ 0.5. When the echo received enters the software subsystem via the gigabit Ethernet link, a frame with complex type data is received. With the initial frame, Stages 2 and 3 of the code generation and matched filters are programmed. In the proposed design, it is indicated that there must be two filters for each channel (float type). However, as complementary pulses are sent in series, the coefficients of the matched filters are correlated for both code1 and code2 having correct and false code responses. To solve this problem, two matched filters were programmed, one with code1 as coefficients and the other with code2 with the function “correlate_and_sync_cc”. These only allow complex type data input and output, which means there are four matched filters with float type allowed to have all of the correct and wrong answers of both filters from both Channel I and Channel Q. To return to a single data frame with only the expected responses, a selective function is generated in Stage 4 through the GNU-Radio out-of-tree (OOT) module with three data inputs: two for the complex frames of the matched filter outputs and one for control (the signal control_1 of Figure 13 is used). In addition to this, since the used daughterboards do not have input filters, root raised cosine (RRC) filters of 128 coefficients are programmed into the matched filters blocks.

**Figure 17:**
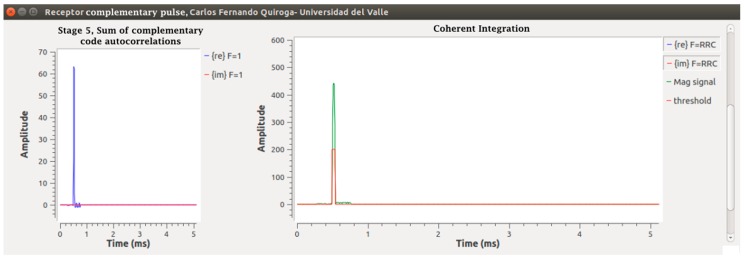
Output Stages 4–8 of the digital receiver without noise.

**Figure 18:**
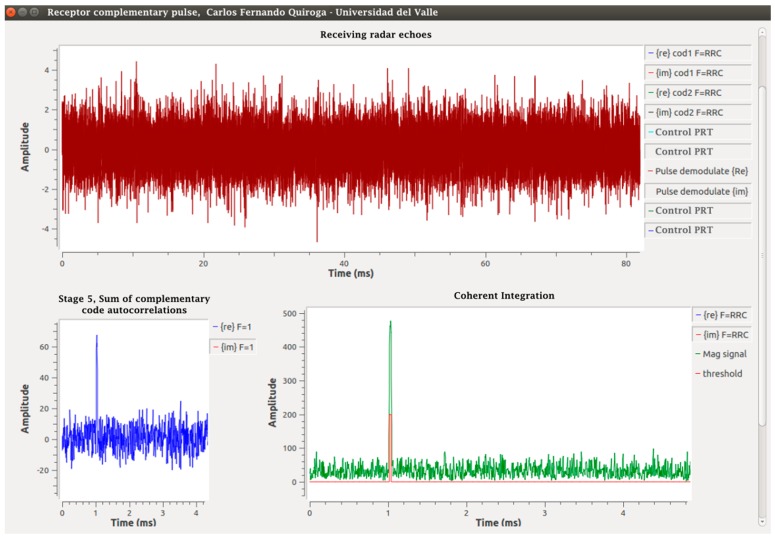
Receiver results with input and echoes with noise.

With the output of the matched filters in an IQ frame, Pulses 1 and 2 are added in Stage 5. Since these responses are in a single data frame, the delay function and an adder are used. The output of this function is a pulse as shown in Figure 4. Stage 6 is the coherent integration of pulses. For this, a function called “integration” is programmed through the “Hierarchical Block” class of GNU-Radio in which the same technique of the previous stage is used with the difference that K − 1 delay functions are programmed in parallel, each with a successive multiple of the first delay and an addition function with K inputs and an output, which integrates all of the K pulses leaving the result in the initial position; an integration with K = 7 was done. Following this, a change of rectangular to polar coordinates was done through the “complex_to_mag” function, corresponding to Stage 7. With the magnitude, the frame enters the “Threshold” function in Stage 8, which is responsible for drawing a pulse if any of its values exceed the proposed V_T_ level. Finally, a concatenation of the control data and the radar pulses is done in a data frame stored in extension .dat ready for post-processing. The results are shown in Figure 17 and Figure 18, from the simulated radar pulses with and without Gaussian noise, to corroborate the detection capacity of the receiver.

### 5.3. Transmitter-Receiver Coupling

The test performed to articulate the transmitter and the receiver consists of a direct physical coupling between these two stages of the ionosonde. This is possible because of the nature of the daughterboards [2]. In order to see the behavior of the system, this allows taking the phase-modulated pulses that have been sent by the transmitter (see Figure 15) and demodulating and processing them [29]. It is important to emphasize that the sources and sinks of the USRP existing in its programming must be synchronized at all times through the “UHD interface”.

**Figure 19:**
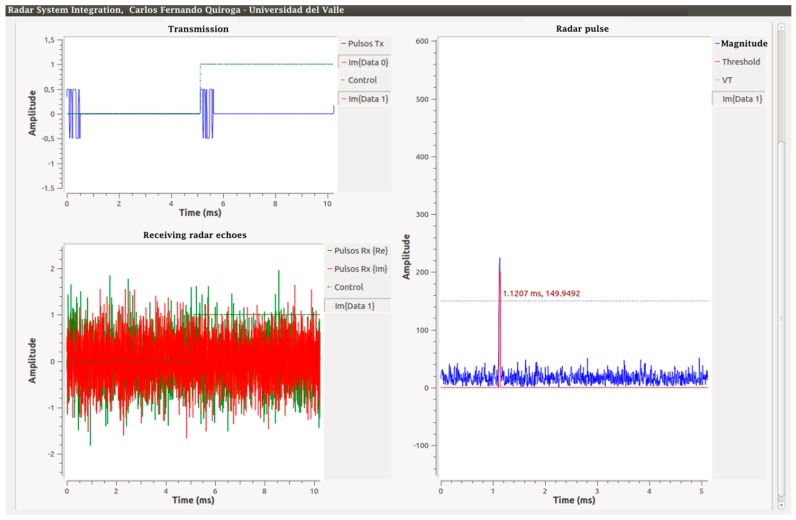
Integration of the radar system.

From this process, a successful demodulation and processing is obtained, shown in Figure 19. However, there is a constant phase shift of 96 μs for all received pulses at the receiver, which must be taken into account when analyzing the data.

**Figure 20:**
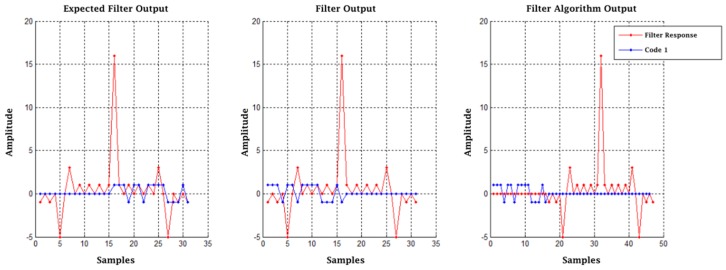
Matched filter outputs.

For the radar integration test, the parameters used were the output amplitude of the pulse interface 0.5, SNR = 0 dB of the reception in A (see Figure 11), and the threshold level V_T_ = 150. An aspect that stands out in this result is the time in which the detection pulse originates in the graph “Radar pulse” in Figure 19, equivalent to approximately 1.12 ms. However, what would be expected is that it is equivalent to the delay obtained previously of 96 μs. This difference is the result of the output of the algorithm implemented for the matched filters, which shows that their response is 128 additional samples after the original 127 samples of the original delay, giving a total delay of 257 samples. Figure 20 explains the output of this algorithm with Code 1 and with one sample per signal (SPS).

If the sampling frequency, the delays produced by the filters and the initial delay are taken into account, the resulting error can be corrected when calculating *h_v_*.

## 6. Tests and Discussion

The results of the previous sections demonstrated a good ability of the USRP N200 to be programmed as a transmitter to generate the shape of the radar pulses with a PRF_1_ and as a receiver to capture and process the echo information from the ionosphere.

### 6.1. Experimental Structure

The frame that is generated after the threshold function (see Figure 16) is ready to be processed. These data are captured and written to a .dat file using the class “file_sink”. The post-processing program used was MATLAB.

A complete system experiment was proposed, which consists of two stages. The first stage is the basis of the experiment and is where echo delays from the ionosphere, wave attenuation per trip and noise are simulated. The experiment is performed with a constant modulation frequency since its variation only affects the amplitude of the signal [2]. It is understood that, with the echo delay simulation, it is possible to simulate the frequency sweep of 1–20 MHz implemented in the transmitter, as each change in frequency represents a certain phase shift. This delay was digitally programmed in the transmission stage using the “delay” class.

If the sampling frequency F_Sa_ is considered, each delayed sample is equivalent to 4 μs, which corresponds to a pulse trip of 1200 m in the atmosphere. Considering this, the minimum delay to be applied in the receiver is of 167 samples if it is estimated that the first layer is found at 100.2 km.

**Figure 21:**
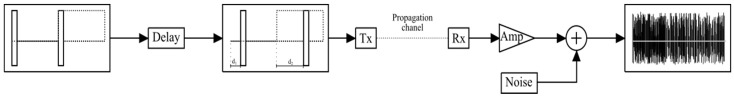
Outline of the proposed experiment, first stage.

The wave and noise attenuation are simulated in the receiver stage. The proposed attenuation of 130 dB is not considered due to the configuration of the experiment, and only the attenuation produced in the channel is taken, which is amplified 11 times to have the system normalized. Noise is simulated by adding to the received signal IQ in A (see Figure 11) Gaussian white noise with the class “noise_source_c” of the same amplitude, or greater than the received signal, to have an SNR ≤ 0. Figure 21 shows an outline of the experiment and the variation of the pulse as it goes through each one of the stages. 

The second stage depends on the binary file saved from GNU Radio Companion (GRC), which contains the information of the pulses detected by the threshold function and the control signal Control_1 of Figure 13. This file is converted to a working format in MATLAB. The function in MATLAB contains an algorithm to find the offset, which is based on detecting the cardinal of each pulse encountered and subtracting the width of the number of changes of state of the control signal. This difference is the gap produced between transmission and reception plus any processing errors detailed above. Once this difference is obtained, the respective mathematical operations are performed to pass it from the number of samples to a height *h_v_*, which are subsequently plotted generating the first approach to an ionogram.

### 6.2. Measurement of the Height of the Ionosphere

**Figure 22:**
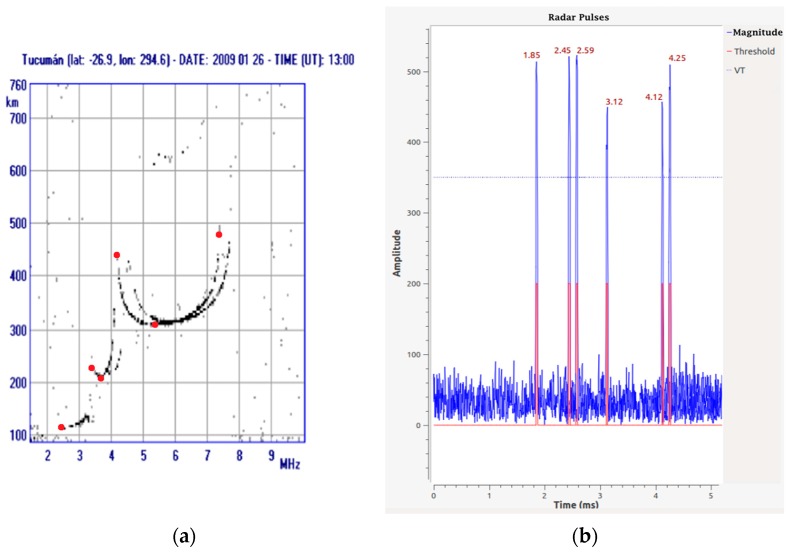
(**a**) Daytime ionogram of San Miguel de Tucumán, Argentina; (**b**) results obtained in the first stage of the experiment.

**Figure 23:**
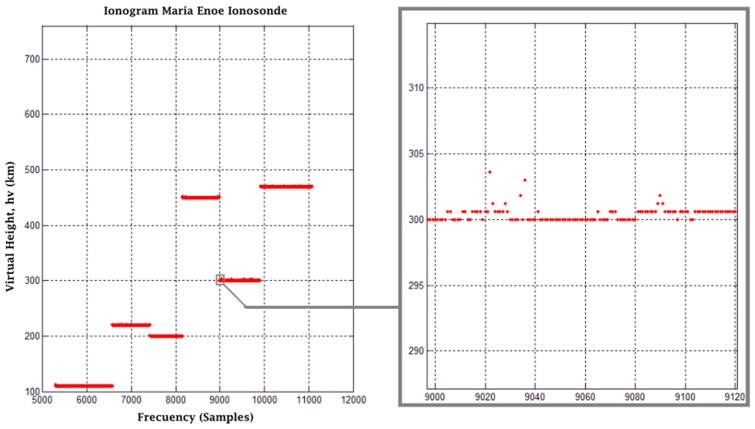
Final results of post-processing, Test 1.

For the experiment, three tests were proposed, each with a different modulation frequency and offsets corresponding to the main heights seen in real ionograms. The first test was performed based on a diurnal ionogram of the digital ionosonde from San Miguel de Tucumán, Argentina, shown in Figure 22a. The red dots represent the most distinctive data of the ionogram. Once the data were identified, the first stage of the experiment was carried out with a modulation frequency of 4 MHz, an output amplitude of 0.5 in the interface, a gain in the receiver of 11 times and an SNR = 0 dB. Figure 22b shows the results obtained (the pulses are superimposed on the same graph; however, it must be understood that not all occur at the same time). The numbers over each pulse are the time in milliseconds of the echo offset with respect to the transmitted pulse. If the representative points in the Tucuman ionogram are followed in order, the result obtained from the pulse arrival corresponds to 1.85, 2.59, 2.45, 4.12, 3.12 and 4.25 ms. Figure 23 shows the result of the second stage of post-processing where a first approach to an ionogram is obtained, representing the heights calculated from the echoes captured at the receiver. The horizontal axis is the representation of a frequency sweep, while the vertical axis shows the virtual height *h_v_* corresponding to the characteristic points already mentioned. The graph on the right shows an approach in which discrete samples are shown for each “frequency”.

**Figure 24:**
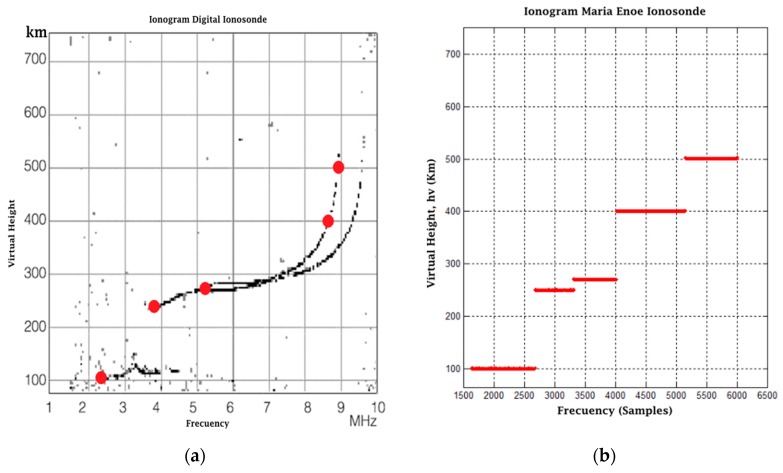
(**a**) Digital ionosonde ionogram; (**b**) final result post-processing Test 2.

**Figure 25:**
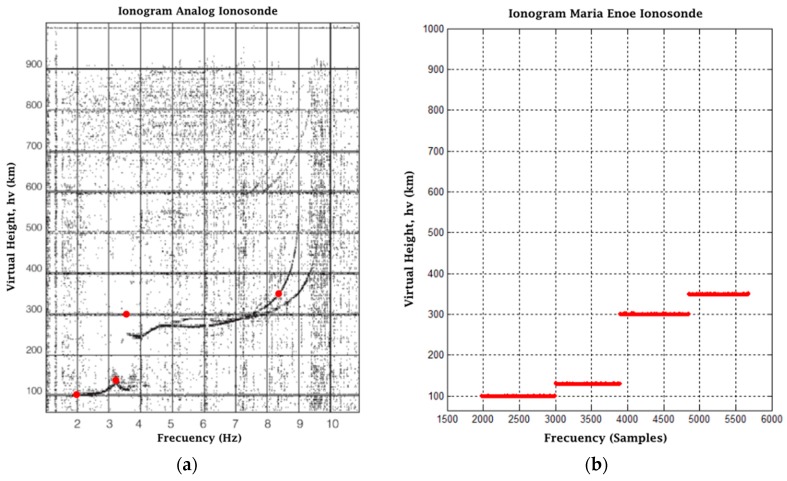
(**a**) Analog ionosonde ionogram; (**b**) Final result post-processing of Test 3.

The results of Tests 2 and 3 are based on the ionograms of a digital ionosonde and an analog ionosonde [23]. Similarly to Test 1, we chose the most representative values of the ionograms, and the expected results are based on them. The tests were performed with a modulation frequency of 2 MHz and 6 MHz, respectively, as well as a transmission amplitude of 0.5 in interface, a gain in the receiver of 11 times and an SNR = 0. The results are presented in Figure 24a,b and Figure 25a,b.

The tests of experiment one demonstrate that the transmitter and receiver programming meet the desired characteristics and that the post-processing is able to print the heights of the ionosphere for the designed range.

## 7. Conclusions

This paper presents the conception and implementation of a small electronic ionosonde prototype, using SRD technology and the USRP N200 radio communication equipment.

The transmitter, receiver and signal processing structures were designed and implemented based on a technical review of both the physical properties of the atmosphere and signal processing techniques, including hardware and software components.

Improving the SNR reduces the transmission power to a maximum of 400 W when using programming techniques.

The pulse compression applied in this development is a direct cause in the reduction of transmission power. The complementary codes used for this technique are the ones that provided better results in terms of PSL.

In the implementation of the matched filter, a gain of twice of that which was expected is achieved, which is sufficient to make a distinction between the received echoes and the unwanted signals.

The coherent integration algorithm that was used is performed in the time domain demonstrating an increase in the SNR of the signal by at least K-times.

The algorithms programmed both in GNU-Radio and MATLAB, in conjunction with the USRP system, showed good performance that was reflected in the increase of the SNR, distinguishing the radar pulses and increasing the detection probability.

The USRP system, together with the basic Tx and Rx boards, has the ability to generate the pulses required at the frequencies needed to scan the ionosphere and receive them to analyze the data. This also confirms that basic Rx and Tx daughterboards are a good choice because they provide enough bandwidth and support information refinement.

A filter that attenuates the high frequency components of the signal was programmed in the pulse modulation stage, which improves the modulation by binary phase code, decreasing the intersymbol interference and optimizing the performance of the matched filters.

The resolution associated with the bandwidth used is approximately 5 km, but this can be improved, taking advantage of the fact that the daughterboards used in the USRP allow a higher sampling frequency than the one used in the design of this experiment.

The system developed together with the programming is able to print the heights of the ionosphere for the designed range.

A high performance is required of the processors because the USRP system requires a gigabit Ethernet connection and a high-speed CPU, which was not available at the laboratory, so the computer system used is insufficient, causing loss of data both in sending and receiving information frames, hampering the USRP-host synchronization task and processing of the signal.

According to the results obtained, the ionosonde implementation by USRP can obtain similar or better precisions than the previous technologies, having the advantage of the greater flexibility of SDR systems and their ease of development in so-called radar software.

## 8. Future Works

Developing a functional ionosonde prototype is a difficult goal to meet. This project takes the first steps and sets the bases to achieve a good development based on USRP technology. The following future works are suggested:

It is necessary to extrapolate the radar programming to another USRP, to have a transmitting device and another receiver, with the objective of developing oblique ionosondes. In addition to this, both devices must be synchronized in order to obtain the ionospheric profiles.

It is necessary to rethink the design of the transmitted pulses to reduce the power even more. Although they worked successfully in the laboratory, it was not taken into account that there are echoes that bounce more than once in the ionospheric layers and would be out of the calculated range. Likewise, decreasing the PRF will allow more time for processing all of the data, balancing this time with the time of generation of the ionogram.

The current version of the proposed prototype is not in real-time because part of the processing is done offline in MATLAB. This does not allow exploiting the true power to the maximum in terms of GNU-Radio processing. For this, it is advisable to program our own post-processing blocks for signals using Octave, which will expand the capacities of the radar and will be able to generate real-time processing.

Implement the matched filters by means of the Out Of Tree (OOT)-module in the frequency domain, since in the time domain, there is the latent possibility of loss of information from the signal.

Explore programming of the internal FPGA by means of some programming language, like Verilog or VHDL.

Although GNU-Radio allows the processing of the signals in the host, part of it could be performed in the FPGA available in the USRP.

Investigate more about the Barker codes and Legendre codes to test them, as they allow easier processing of the signals.

Do a more detailed analysis and development of the amplification stage, power and antennas of the transmitter and receiver, since they are important parts of the prototype.

## Figures and Tables

**Table 1:** Ionosonde specifications.

Parameter	Requirement
Range of Height (Min–Max) [14]	90−750 km
Resolution [24]	5 km
Frequency Range	1−20 MHz
Step	50 kHz
Very High Loss	(Rx power)/(Tx power) = −130 dB
Scan duration (max)	3 min at 50-kHz steps
Max. pulse power Tx	600 W
Input Spurious-free dynamic range (SFDR)	80 dB
*P_FA_*/*P*_D_	10^−6^/0.5

**Table 2:** Preliminary design parameters of the transmission signal.

Parameter	Pre-Calculated Value
Bandwidth B	30 kHz
*t_s_*	33.3 µs
No. Bits (Complementary Code)	18
τ	600 µs
PRF_1_/PRT_1_	200 Hz/5 ms

**Table 3:** Power of ionosonde designs around the world.

Ionosonde	Maximum Pulse Power (W)
AIS-INGV Ionosonde [14]	250
Umass Lowell Space Science lab. Digisonde [25]	300
The Canadian Advanced Digital Ionosonde [26]	600
Ionosonde [16]	400

**Table 4:** Transmission signal design parameters.

Parameter	Value
ts	32 µs
No. Bits (Complementary Code)	16
τ	512 µs
PRT_1_	5.12 ms
PRT_2_	10.24 ms

**Table 5:** Specifications of the designed ionosonde.

Parameter	Value
Height range	76.8–768 km
Resolution	4.8 km
Frequency range	1–20 MHz
Step	50 kHz
Scan duration (max.)	39 s
Max. pulse power Tx	400 W
Number of samples	144
Number of soundings	381
Sampling frequency	250 kHz
Samples per symbol (SPS)	8
